# Supplementary material for: The Systems Biology Research Tool: evolvable open-source software
Source: BMC Syst Biol. 2008 Jun 29;2:55. doi: 10.1186/1752-0509-2-55 (PMC2446383; doi:10.1186/1752-0509-2-55)
Supplement: Additional file 1 — SBRT Archive. An archive of the current version of the Systems Biology Research Tool. [file 1752-0509-2-55-S1.zip › sbrt-1.4.0/doc/developers_guide/api/sbrt/shell/io/ProcessInputFile.html]

ProcessInputFile


|  |  |  |
| --- | --- | --- |
| **Overview** \| **Package** \| **Class** \| **Use** \| **Tree** \| **Deprecated** \| **Index** \| **Help** | |  |
| **PREV CLASS**   **NEXT CLASS** | **FRAMES**    **NO FRAMES**     **All Classes** |  |
| SUMMARY: NESTED \| FIELD \| CONSTR \| METHOD | DETAIL: FIELD \| CONSTR \| METHOD |  |


---


## sbrt.shell.io Class ProcessInputFile

```
java.lang.Object
  sbrt.shell.io.AbstractMapInputFile<K,V>
      sbrt.shell.io.RomiFile<java.lang.String,java.lang.String>
          sbrt.shell.io.ProcessInputFile
```

**All Implemented Interfaces:**: java.lang.Iterable<java.util.Map<java.lang.String,java.lang.String>>, InputFile<java.util.Map<java.lang.String,java.lang.String>>, MapInputFile<java.lang.String,java.lang.String>, MapTextFile<java.lang.String,java.lang.String>, MapTextInputFile<java.lang.String,java.lang.String>, SbrtFile, ManagerInput<java.lang.String,java.lang.String>

---

``` public final class ProcessInputFile extends RomiFile<java.lang.String,java.lang.String> implements ManagerInput<java.lang.String,java.lang.String> ```

This class is used to represent process input files.
These are the files that can be given as a single command line
argument to sbrt.
The are used to specify all the details required to construct
a process using a process manager.

**Author:**
:   This class was written and documented by
    Jeremiah Wright while in the Wagner lab.

---

**Constructor Summary**

`ProcessInputFile(java.lang.String fileName)`
  
          Constructs a new process input file from the
provided argument.

`ProcessInputFile(java.lang.String fileName,
AppInputFileLineFormat lineFormat)`
  
          Constructs a new process input file from the
provided arguments.


| **Method Summary** | |
| --- | --- |
| `java.lang.String` | `getName()`             Returns the canonical name of this file. |
| `java.lang.String` | `getOptionalValue(java.lang.Object key)`             Returns the value corresponding to the provided key. |
| `java.lang.String` | `getRequiredValue(java.lang.Object key)`             Returns the value corresponding to the provided key. |
| `boolean` | `hasValueFor(java.lang.Object key)`             Indicates if a value exists for the provided key in this manager input. |
| `java.util.Map<java.lang.String,java.lang.String>` | `toMap()`             Returns the information contained in this file as a map. |
| `java.lang.String` | `toString()`             Returns a string representation of the information contained in this file. |

**Methods inherited from class sbrt.shell.io.RomiFile**

`getKeys, getMap, iterator`

**Methods inherited from class sbrt.shell.io.AbstractMapInputFile**

`close, getCanonicalName, getMapFormat, getOriginalName, getRequiredKeys, isClosed, lineIterator`

**Methods inherited from class java.lang.Object**

`clone, equals, finalize, getClass, hashCode, notify, notifyAll, wait, wait, wait`

**Constructor Detail**

### ProcessInputFile

```
public ProcessInputFile(java.lang.String fileName,
                        AppInputFileLineFormat lineFormat)
```

:   Constructs a new process input file from the
    provided arguments.

    **Parameters:**: `fileName` - the name of the file to be read.: `lineFormat` - the format used to parse its lines.

---


### ProcessInputFile

```
public ProcessInputFile(java.lang.String fileName)
```

:   Constructs a new process input file from the
    provided argument.

    **Parameters:**: `fileName` - the name of the file to be read.


**Method Detail**

### toMap

```
public java.util.Map<java.lang.String,java.lang.String> toMap()
```

:   Returns the information contained in this file as a map.

    :   **Specified by:**: `toMap` in interface `ManagerInput<java.lang.String,java.lang.String>`
    :   **Returns:**: keywords mapped to their respective values.

---


### getName

```
public final java.lang.String getName()
```

:   Returns the canonical name of this file.

    :   **Specified by:**: `getName` in interface `ManagerInput<java.lang.String,java.lang.String>`
    :   **Returns:**: the canonical name of this file.

---


### hasValueFor

```
public boolean hasValueFor(java.lang.Object key)
```

:   Indicates if a value exists for the provided key
    in this manager input.

    :   **Specified by:**: `hasValueFor` in interface `ManagerInput<java.lang.String,java.lang.String>`
    :   **Parameters:**: `key` - the key whose presence in this input is to be tested. **Returns:**: `true` if this input contains a value for the specified key.

---


### getOptionalValue

```
public java.lang.String getOptionalValue(java.lang.Object key)
```

:   Returns the value corresponding to the provided key.

    :   **Specified by:**: `getOptionalValue` in interface `ManagerInput<java.lang.String,java.lang.String>`
    :   **Parameters:**: `key` - the key whose value is to be returned. **Returns:**: the value corresponding to the provided key, or `null` if the value does not exist.

---


### getRequiredValue

```
public java.lang.String getRequiredValue(java.lang.Object key)
```

:   Returns the value corresponding to the provided key.

    :   **Specified by:**: `getRequiredValue` in interface `ManagerInput<java.lang.String,java.lang.String>`
    :   **Parameters:**: `key` - the key whose value is to be returned. **Returns:**: the value corresponding to the provided key. **Throws:**: `ApplicationException` - if the value does not exist.

---


### toString

```
public java.lang.String toString()
```

:   Returns a string representation of the information contained
    in this file.

    :   **Overrides:**: `toString` in class `java.lang.Object`
    :   **Returns:**: a string representation of the information contained in this file.


---


|  |  |  |
| --- | --- | --- |
| **Overview** \| **Package** \| **Class** \| **Use** \| **Tree** \| **Deprecated** \| **Index** \| **Help** | |  |
| **PREV CLASS**   **NEXT CLASS** | **FRAMES**    **NO FRAMES**     **All Classes** |  |
| SUMMARY: NESTED \| FIELD \| CONSTR \| METHOD | DETAIL: FIELD \| CONSTR \| METHOD |  |


---
